# Isomorphous diethyl 1-(4-chloro­benz­yl)-4-(4-chloro­phen­yl)-2,2-dioxo-3,4,6,7,8,8a-hexa­hydro-1*H*-pyrrolo­[2,1-*c*][1,4]thia­zine-1,3-di­carboxyl­ate and its 1-(4-methyl­benz­yl)-4-(4-methyl­phen­yl)-substituted analogue obeying the chloro–methyl exchange rule

**DOI:** 10.1107/S2056989018011416

**Published:** 2018-08-16

**Authors:** R. Sribala, N. Srinivasan, S. Indumathi, R. V. Krishnakumar

**Affiliations:** a Department of Physics, Thiagarajar College, Madurai 625 009, India; b School of Chemistry, Madurai Kamaraj University, Madurai 625 021, India

**Keywords:** crystal structure, pyrrolo derivatives, thia­zine, hydrogen bonding, Hirshfeld surface analysis

## Abstract

Accurate studies on the effect of substituents on the crystal packing are essential for understanding the inter­molecular inter­actions and thus paving the way to crystal structure prediction. The crystal structures of diethyl 1-(4-chloro­benz­yl)-4-(4-chloro­phen­yl)-2,2-dioxo-3,4,6,7,8,8a-hexa­hydro-1*H*-pyrrolo­[2,1-*c*][1,4]thiazine-1,3-di­carboxyl­ate, C_26_H_29_Cl_2_NO_6_S, (I), and its isomorphous pair diethyl 1-(4-methyl­benz­yl)-4-(4-methyl­phen­yl)-2,2-dioxo-3,4,6,7,8,8a-hexa­hydro-1*H*-pyrrolo­[2,1-*c*][1,4]thia­zine-1,3-di­carboxyl­ate, C_28_H_35_NO_6_S, (II), are described. The mol­ecular aggregation patterns appear to be strikingly similar despite changes in the substituents, with a Cl atom in (I) being replaced by a methyl group in (II). Inspite of the chemical modifications, the structures of (I) and (I) are isomorphous, isostructural and found to obey the chlorine–methyl exchange rule. Both the structures feature C—H⋯O hydrogen bonding. However, a distinguishing feature between (I) and (II) is observed in the conformation of the pyrrole rings where the twist occurs on different C—N bonds. Hirshfeld analysis of both structures is presented and discussed.

## Abstract 2

The influence of the substituents in the crystals of the title compounds has not made any significant effect on the crystal packing and inter­molecular hydrogen bonds. The validity of chlorine–methyl exchange rule is confirmed.

## Chemical context   

Crystal structure determinations of small mol­ecules have often revealed inter­esting features that have direct relationships to their predicted structures. In this context, the display of chlorine–methyl and benzene–thio­phene exchange rules in the close-packing model of organic mol­ecules (Kitaigorodskii, 1973 ▸) may be regarded as crucial to crystal engineering studies. In the present study, the crystal structures of two closely related heterocyclic analogues which differ only by a chlorine-methyl substituent, *viz*. diethyl 1-(4-chloro­benz­yl)-4-(4-chloro­phen­yl)-2,2-dioxo-3,4,6,7,8,8a-hexa­hydro-1*H*-pyrrolo[2,1-*c*][1,4]thia­zine-1,3-di­carboxyl­ate (I) and its isomorphous pair diethyl 1-(4-methyl­benz­yl)-4-(4-methyl­phen­yl)-2,2-dioxo-3,4,6,7,8,8a-hexa­hydro-1*H*-pyrrolo­[2,1-*c*][1,4]thiazine-1,3-di­carboxyl­ate (II) have been determined. Inter­estingly, (I) and (II) are found to obey the chorine–methyl exchange rule and hence are isomorphous and isostructural. While there is evidence that the Cl–Me rule based solely on the size of the substituent need not always be valid (Jones *et al.*, 1981 ▸; Gnanaguru *et al.*, 1984 ▸), it has been observed as a valid proposition for large, irregularly shaped mol­ecules (Desiraju & Sarma, 1986 ▸). Although crystal-packing inter­actions in large irregularly shaped mol­ecules such as (I) and (II) are not entirely based on geometrical considerations, the role of inter­molecular inter­actions in such pairs of structures seems far from being complex with striking similarities involving the strongest among them. In some of our earlier structure determinations to ascertain the validity of exchange rules, two obeying the chloro–phenyl exchange (Rajni Swamy *et al.*, 2013 ▸; Rajni Swamy, 2016 ▸) and another obeying the benzene–thio­phene exchange (Rajni Swamy, 2016 ▸) have been observed.
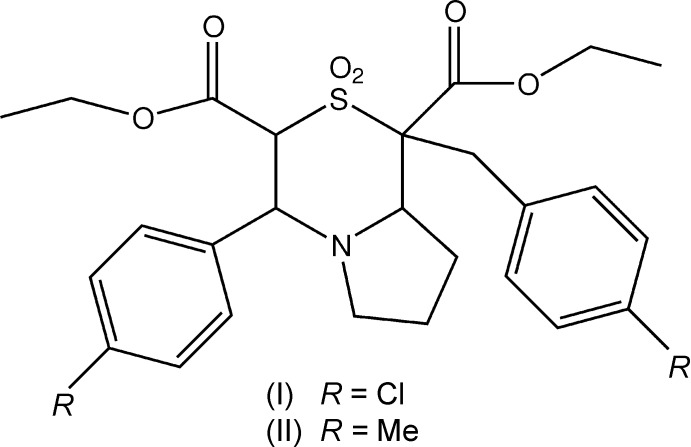


Both (I) and (II) are thia­zine derivatives that may potentially exhibit pharmacological activities in view of the presence of nitro­gen and sulfur atoms as constituents of the fused pyrrolo­thia­zine ring (Moriyama *et al.*, 2004 ▸; Koketsu *et al.*, 2002 ▸; Rai *et al.*, 2013 ▸). Derivatives of thia­zine have been shown to exhibit calcium antagonist activities (Erker, 1998 ▸) and various inhibitory activities on central nervous system (Grandolini *et al.*, 1997 ▸; Malinka *et al.*, 2002 ▸). Pyrrolo­thia­zine derivatives have been employed as anti-inflammatory, anti-fungal and anti-microbial agents (Armenise *et al.*, 1991 ▸; Armenise *et al.*, 1998 ▸). The present work reports the detailed description of the crystal structures of (I) and (II) along with Hirshfeld surface analysis of their respective inter­molecular inter­actions.

## Structural commentary   

**Figure 1:**
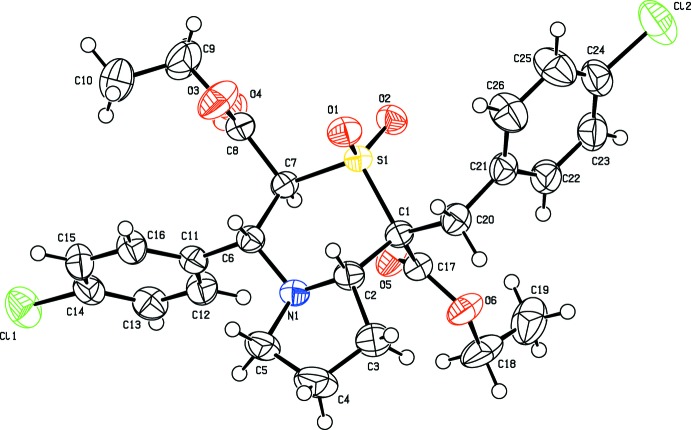
Displacement ellipsoid plot (50% probability level) of title compound (I), showing the atom-labelling scheme.

The mol­ecular structures of the title compounds differ from each other only by a chlorine atom in (I) being replaced by a methyl group in (II). The replacement has not effected changes in their unit-cell parameters, lattice type and space group, indicating that structures (I) and (II) are isomorphous in nature (Figs. 1 ▸ and 2 ▸). The pyrrolo ring (N1/C2–C5) in compound (I) adopts a twisted conformation on N1—C2 with puckering parameters *Q*(2) = 0.3604 (19) Å and φ = 191.2 (4)°. However, in compound (II) the twisted conformation is observed on the C5—N1 bond with *Q*(2) = 0.377 (2) Å and φ(2) = 169.3 (4)°. The Cremer and Pople puckering parameters of the six-membered heterocyclic ring in (I) are *Q* = 0.6441 (15) Å, θ = 8.51 (14)° and φ = 95.8 (8)°, close to a chair conformation (^1^ *C*_4_), which is comparable with the values of *Q* = 0.6511 (16) Å, θ = 9.53 (15)° and φ = 97.5 (7)° for (II). The dihedral angle between the planes of the thia­zine and pyrrolo rings is 6.68 (10)° in compound (I) compared with 8.06 (11)° in (II). Similarly the thia­zine ring and the chloro-substituted benzyl ring (C21–C26) in (I) subtend a dihedral angle of 78.61 (9)° [79.48 (9)° for the methyl-substituted benzyl ring (II)]. The terminal methyl carbon atom C10 deviates from the plane involving the carboxyl group (C7/C8/O3/O4/C9) by 1.371 (3) Å in compound (I) and 1.409 (3) Å in compound (II). Similarly the methyl­carbon atom C19 deviates from the C1/C17/O5/O6/C18 plane by 1.246 (3) Å in (I) and 1.203 (3) Å in (II). The dihedral angles between these two planes are 12.73 (10) and 12.07 10)° in compounds (I) and (II), respectively.

## Supra­molecular features   

**Figure 3:**
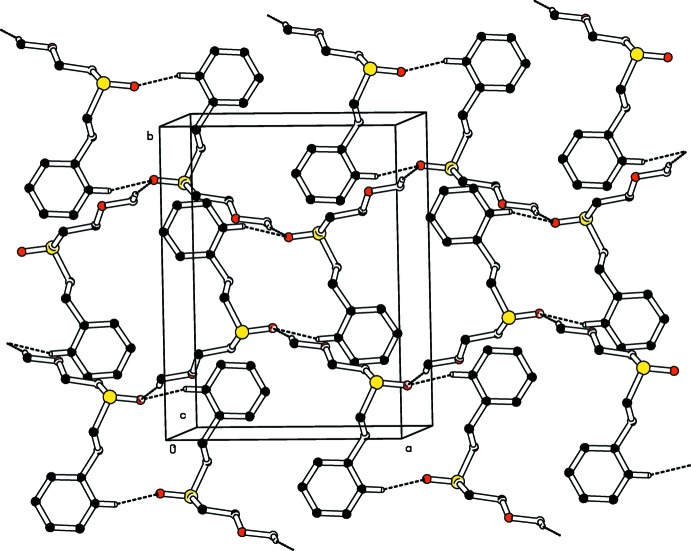
Part of the crystal structure of compound (I), showing the formation of an 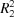
(14) ring. Dashed lines indicate hydrogen bonds. H atoms not involved in the hydrogen bonding have been omitted for the sake of clarity.

The crystal packing of both compounds (Figs. 3 ▸ and 4 ▸) features C—H⋯O hydrogen bonding (Tables 1 ▸ and 2 ▸) and π–π inter­actions. The C—H⋯O inter­actions, which are similar in strength and geometry, involve only one of the two dioxo oxygen atoms, *viz*. O1. The non participation of the other oxygen atom (O2) cannot be explained from the viewpoint of inter­molecular inter­actions whereas the absence of such inter­actions involving O3 and O5 may be attributed to steric factors arising from an unfavourable packing geometry. In both crystals, mol­ecules are connected into inversion dimers *via* pairs of weak C—H⋯O hydrogen bonds, forming 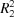
(14) graph-set motifs. These dimers are further connected *via* weak C—H⋯O inter­actions into chains running along [011]. A parallel-displaced π–π stacking inter­action is observed in both compounds between the C21–C26 benzyl rings. In (I), *Cg*⋯*Cg*(1 − *x*, −*y*, 2 − *z*) = 4.0485 (13) Å, with a slippage of 1.749 Å [for (II), *Cg*⋯*Cg*(1 − *x*, 2 − *y*, 2 − *z*) = 4.0554 (14) Å, slippage of 1.711 Å] where *Cg* is the ring centroid.

## Hirshfeld Surface Analysis   

**Figure 5:**
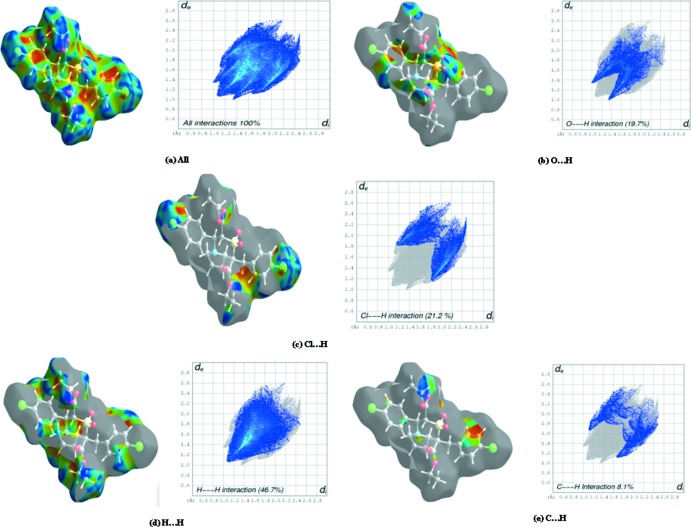
Hirshfeld surface of compound (I) mapped over shape-index and decomposed finger print plots of dominant inter­actions showing (*a*) all, (*b*) O⋯H, (*c*) Cl⋯H, (*d*) H⋯H and (*e*) C⋯H inter­actions.

Hirshfeld surface analysis is a graphical tool to investigate the packing modes and nature of prominent inter­molecular inter­actions in crystal structures. The Hirshfeld surfaces (Spackman & Jayatilaka, 2009 ▸) and the associated two-dimensional fingerprint plots were generated using *CrystalExplorer* 3.0 software (Wolff *et al.*, 2012 ▸). In the present work, the nature of the inter­molecular inter­actions in the two structures is similar because of their isomorphism. The Hirshfeld surfaces mapped with shape-index together with decomposed fingerprint plots for (I) and (II) are shown in Figs. 5 ▸ and 6 ▸, respectively. In both the structures, the mol­ecules participate in weak C—H⋯O hydrogen bonds, which are indicated by red spots on the surface plots. The O⋯H/H⋯O inter­molecular inter­actions appear as distinct sharp spikes in the fingerprint plots. The area between the spikes corresponds to the H⋯H contacts, which account for nearly 46.7% of the surface in (I) and 70.6% in (II). The Cl⋯H/H⋯Cl inter­action, shown by two wing-like projections in (I), is obviously absent in (II). The Hirshfeld surfaces of the two compounds show striking similarities in the relative contributions of the inter­actions and a noteworthy difference, accounted for by the presence of Cl⋯H/H⋯Cl inter­actions in (I) and their absence in (II).

## Database survey   

A search in the Cambridge Structural Database (CSD Version 5.39, update November 2017; Groom *et al.*, 2016 ▸) for the skeleton of the title compound without chlorine or methyl substitution for which 3D coordinates were determined with no disorder, no ions and no other errors, with *R* factors less than 0.05 revealed only one structure, with refcode EXIYAM (Chitradevi, *et al.*, 2011 ▸). A search on 4-thio­morpholine-1,1-dione gave five hits with refcodes EXIYAM, IDOGIT (Chitradevi *et al.*, 2013 ▸), IJULAB (Sugumar *et al.*, 2011 ▸), NEVCUN (Indumathi *et al.*,2007 ▸) and ZEXYEG (Krishnaiah *et al.*, 1995 ▸).

## Synthesis and crystallization   

A mixture of ethyl 2-[(2-eth­oxy-2-oxo-eth­yl)sulfon­yl]acetate (1.6 mmol), aromatic aldehyde (3.2 mmol) and pyrrolidine (1.6mmol) was dissolved in ethanol (10 mL), heated until the solution turned yellow and stirred at room temperature for 2–5 days. After completion of the reaction, the crude product was purified using flash column chromatography on silica gel (230–400 mesh) with petroleum ether and ethyl acetate mixture (95:5 *v*/*v*) as eluent (Indumathi *et al.*, 2007 ▸).

## Refinement   

Crystal data, data collection and structure refinement details are summarized in Table 3 ▸. In both compounds, the carbon-bound H atoms were placed in calculated positions (C—H = 0.93–0.97 Å) and were included in the refinement in the riding-model approximation, with *U*_iso_(H) set at 1.2–1.5*U*_eq_(C).

## Supplementary Material

Crystal structure: contains datablock(s) I, II, global. DOI: 10.1107/S2056989018011416/xu5934sup1.cif

Structure factors: contains datablock(s) I. DOI: 10.1107/S2056989018011416/xu5934Isup2.hkl

Structure factors: contains datablock(s) II. DOI: 10.1107/S2056989018011416/xu5934IIsup3.hkl

Supporting information file. DOI: 10.1107/S2056989018011416/xu5934Isup4.cml

Supporting information file. DOI: 10.1107/S2056989018011416/xu5934IIsup5.cml

CCDC references: 1861314, 1861313

Additional supporting information:  crystallographic information; 3D view; checkCIF report

## Figures and Tables

**Figure 2:**
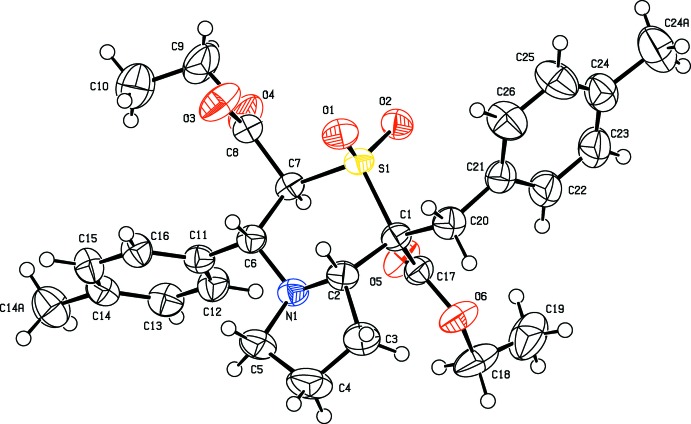
Displacement ellipsoid plot (50% probability level) of title compound (II), showing the atom-labelling scheme.

**Figure 4:**
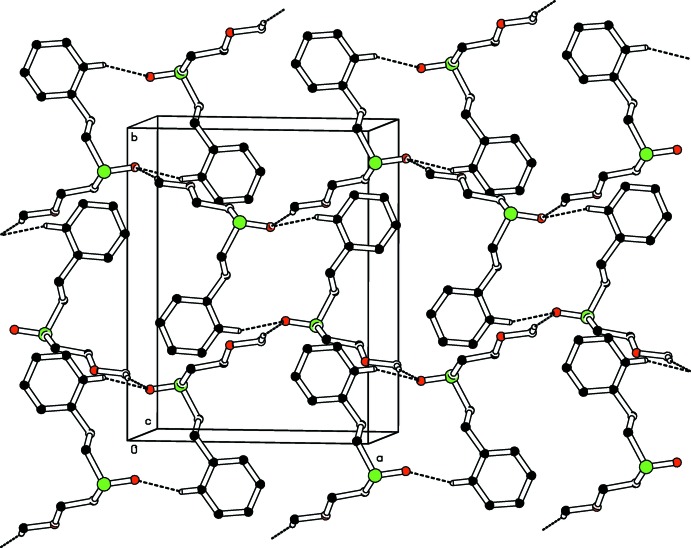
Part of the crystal structure of compound (II), showing the formation of an 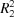
(14) ring. Dashed lines indicate hydrogen bonds. H atoms not involved in the hydrogen bonding have been omitted for the sake of clarity.

**Figure 6:**
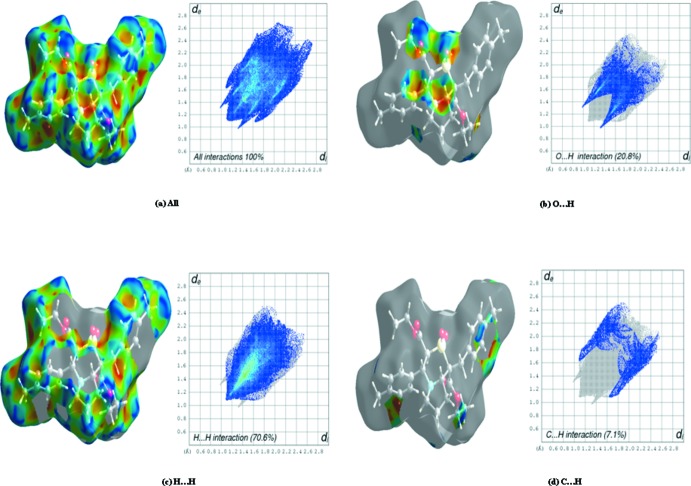
Hirshfeld surface of compound (II) mapped over shape-index and decomposed finger print plots of dominant inter­actions showing (*a*) all, (*b*) O⋯H, (*c*) H⋯H and (*d*) C⋯H inter­actions.

**Table 1:** Hydrogen-bond geometry (Å, °) for (I)

*D*—H⋯*A*	*D*—H	H⋯*A*	*D*⋯*A*	*D*—H⋯*A*
C16—H16⋯O1^i^	0.93	2.50	3.335 (2)	149
C18—H18*A*⋯O1^ii^	0.97	2.45	3.397 (2)	166

Symmetry codes: (i) 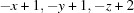; (ii) 
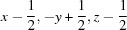
.

**Table 2:** Hydrogen-bond geometry (Å, °) for (II)

*D*—H⋯*A*	*D*—H	H⋯*A*	*D*⋯*A*	*D*—H⋯*A*
C16—H16⋯O1^i^	0.93	2.57	3.406 (2)	150
C18—H18*B*⋯O1^ii^	0.97	2.40	3.333 (2)	161

Symmetry codes: (i) 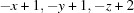; (ii) 
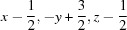
.

**Table 3:** Experimental details

	(I)	(II)
Crystal data		
Chemical formula	C_26_H_29_Cl_2_NO_6_S	C_28_H_35_NO_6_S
*M*_r_	554.46	513.63
Crystal system, space group	Monoclinic, *P*2_1_/*n*	Monoclinic, *P*2_1_/*n*
Temperature (K)	293	293
*a*, *b*, *c* (Å)	11.6596 (4), 14.5734 (4), 15.7000 (5)	11.8641 (5), 14.4765 (6), 15.8654 (7)
β (°)	104.635 (2)	104.960 (2)
*V* (Å^3^)	2581.19 (14)	2632.5 (2)
*Z*	4	4
Radiation type	Mo *K*α	Mo *K*α
μ (mm^−1^)	0.38	0.17
Crystal size (mm)	0.30 × 0.22 × 0.20	0.26 × 0.22 × 0.20

Data collection		
Diffractometer	Bruker SMART APEXII CCD	Bruker SMART APEXII CCD
Absorption correction	Multi-scan (*SADABS*; Bruker, 2009 ▸)	Multi-scan (*SADABS*; Bruker, 2009 ▸)
*T*_min_, *T*_max_	0.858, 1.000	0.863, 1.000
No. of measured, independent and observed [*I* > 2σ(*I*)] reflections	36335, 8631, 5838	34960, 7985, 5294
*R*_int_	0.027	0.032
(sin θ/λ)_max_ (Å^−1^)	0.737	0.713

Refinement		
*R*[*F* ^2^ > 2σ(*F* ^2^)], *wR*(*F* ^2^), *S*	0.047, 0.143, 1.03	0.051, 0.163, 1.03
No. of reflections	8631	7985
No. of parameters	325	325
H-atom treatment	H-atom parameters constrained	H-atom parameters constrained
Δρ_max_, Δρ_min_ (e Å^−3^)	0.37, −0.25	0.36, −0.22

Computer programs: *APEX2* and *SAINT* (Bruker, 2009 ▸), *SHELXS2013* (Sheldrick, 2008 ▸), *SHELXL2018* (Sheldrick, 2015 ▸), *PLUTON* (Spek, 2009 ▸) and *publCIF* (Westrip, 2010 ▸).

## supplementary crystallographic information

### Diethyl 1-(4-chlorobenzyl)-4-(4-chlorophenyl)-2,2-dioxo-3,4,6,7,8,8a-hexahydro-1H-pyrrolo[2,1-c][1,4]thiazine-1,3-dicarboxylate (I) Crystal data

**Table:**

C_26_H_29_Cl_2_NO_6_S	*F*(000) = 1160
*M**_r_* = 554.46	*D*_x_ = 1.427 Mg m^−^^3^*D*_m_ = 1.43 Mg m^−^^3^*D*_m_ measured by floatation method
Monoclinic, *P*2_1_/*n*	Mo *K*α radiation, λ = 0.71073 Å
*a* = 11.6596 (4) Å	Cell parameters from 5292 reflections
*b* = 14.5734 (4) Å	θ = 5.0–57.6°
*c* = 15.7000 (5) Å	µ = 0.38 mm^−^^1^
β = 104.635 (2)°	*T* = 293 K
*V* = 2581.19 (14) Å^3^	Block, colorless
*Z* = 4	0.30 × 0.22 × 0.20 mm

### Diethyl 1-(4-chlorobenzyl)-4-(4-chlorophenyl)-2,2-dioxo-3,4,6,7,8,8a-hexahydro-1H-pyrrolo[2,1-c][1,4]thiazine-1,3-dicarboxylate (I) Data collection

**Table:**

Bruker SMART APEXII CCD diffractometer	5838 reflections with *I* > 2σ(*I*)
Radiation source: fine-focus sealed tube	*R*_int_ = 0.027
φ and ω scans	θ_max_ = 31.6°, θ_min_ = 1.9°
Absorption correction: multi-scan (*SADABS*; Bruker, 2009)	*h* = −17→17
*T*_min_ = 0.858, *T*_max_ = 1.000	*k* = −21→20
36335 measured reflections	*l* = −23→23
8631 independent reflections	

### Diethyl 1-(4-chlorobenzyl)-4-(4-chlorophenyl)-2,2-dioxo-3,4,6,7,8,8a-hexahydro-1H-pyrrolo[2,1-c][1,4]thiazine-1,3-dicarboxylate (I) Refinement

**Table:**

Refinement on *F*^2^	0 restraints
Least-squares matrix: full	Hydrogen site location: inferred from neighbouring sites
*R*[*F*^2^ > 2σ(*F*^2^)] = 0.047	H-atom parameters constrained
*wR*(*F*^2^) = 0.143	*w* = 1/[σ^2^(*F*_o_^2^) + (0.0699*P*)^2^ + 0.608*P*] where *P* = (*F*_o_^2^ + 2*F*_c_^2^)/3
*S* = 1.03	(Δ/σ)_max_ = 0.002
8631 reflections	Δρ_max_ = 0.37 e Å^−^^3^
325 parameters	Δρ_min_ = −0.24 e Å^−^^3^

### Diethyl 1-(4-chlorobenzyl)-4-(4-chlorophenyl)-2,2-dioxo-3,4,6,7,8,8a-hexahydro-1H-pyrrolo[2,1-c][1,4]thiazine-1,3-dicarboxylate (I) Special details

**Table:**

Geometry. All esds (except the esd in the dihedral angle between two l.s. planes) are estimated using the full covariance matrix. The cell esds are taken into account individually in the estimation of esds in distances, angles and torsion angles; correlations between esds in cell parameters are only used when they are defined by crystal symmetry. An approximate (isotropic) treatment of cell esds is used for estimating esds involving l.s. planes.

### Diethyl 1-(4-chlorobenzyl)-4-(4-chlorophenyl)-2,2-dioxo-3,4,6,7,8,8a-hexahydro-1H-pyrrolo[2,1-c][1,4]thiazine-1,3-dicarboxylate (I) Fractional atomic coordinates and isotropic or equivalent isotropic displacement parameters (Å^2^)

**Table:**

	*x*	*y*	*z*	*U*_iso_*/*U*_eq_	
S1	0.32800 (3)	0.34179 (3)	1.03337 (2)	0.03295 (10)	
Cl1	−0.00585 (5)	0.85049 (4)	0.91942 (4)	0.06627 (16)	
Cl2	0.38130 (7)	−0.10569 (4)	1.17052 (5)	0.0853 (2)	
O1	0.45439 (10)	0.35292 (9)	1.05844 (8)	0.0442 (3)	
O2	0.27381 (11)	0.29006 (8)	1.09012 (7)	0.0432 (3)	
O3	0.39736 (13)	0.53038 (12)	1.13297 (10)	0.0674 (4)	
O4	0.20861 (13)	0.52151 (10)	1.13778 (9)	0.0590 (4)	
O5	0.08678 (11)	0.31457 (9)	0.91571 (9)	0.0515 (3)	
O6	0.14113 (12)	0.21823 (10)	0.82265 (8)	0.0532 (3)	
N1	0.26062 (11)	0.45390 (9)	0.86029 (8)	0.0362 (3)	
C1	0.29435 (13)	0.29532 (11)	0.92235 (9)	0.0344 (3)	
C2	0.33387 (15)	0.37130 (11)	0.86656 (10)	0.0390 (3)	
H2	0.416042	0.387925	0.895085	0.047*	
C3	0.3266 (2)	0.34621 (15)	0.77102 (12)	0.0634 (6)	
H3A	0.399936	0.318238	0.765640	0.076*	
H3B	0.261824	0.303885	0.748523	0.076*	
C4	0.3057 (2)	0.43494 (17)	0.72276 (13)	0.0691 (6)	
H4A	0.236877	0.430465	0.673075	0.083*	
H4B	0.373895	0.451028	0.700958	0.083*	
C5	0.28580 (19)	0.50628 (14)	0.78658 (11)	0.0508 (4)	
H5A	0.355794	0.544158	0.806892	0.061*	
H5B	0.219325	0.545392	0.759368	0.061*	
C6	0.28676 (13)	0.50771 (10)	0.94154 (9)	0.0343 (3)	
H6	0.370972	0.524206	0.957456	0.041*	
C7	0.25832 (13)	0.45226 (10)	1.01733 (9)	0.0334 (3)	
H7	0.172299	0.443640	1.004568	0.040*	
C8	0.29875 (16)	0.50547 (11)	1.10326 (10)	0.0411 (3)	
C9	0.2340 (3)	0.57919 (17)	1.21645 (15)	0.0740 (7)	
H9A	0.304941	0.556664	1.257728	0.089*	
H9B	0.168940	0.574461	1.244380	0.089*	
C10	0.2512 (3)	0.67602 (17)	1.19685 (18)	0.0808 (7)	
H10A	0.267583	0.710943	1.250422	0.121*	
H10B	0.316632	0.681308	1.170343	0.121*	
H10C	0.180636	0.699105	1.156995	0.121*	
C11	0.21287 (14)	0.59445 (11)	0.93111 (10)	0.0362 (3)	
C12	0.08995 (15)	0.59070 (13)	0.90414 (12)	0.0467 (4)	
H12	0.052715	0.534480	0.888783	0.056*	
C13	0.02241 (16)	0.66873 (13)	0.89977 (13)	0.0495 (4)	
H13	−0.059830	0.665665	0.881391	0.059*	
C14	0.07868 (16)	0.75169 (12)	0.92311 (11)	0.0440 (4)	
C15	0.19989 (17)	0.75719 (12)	0.94920 (13)	0.0493 (4)	
H15	0.236848	0.813579	0.964160	0.059*	
C16	0.26658 (15)	0.67846 (12)	0.95307 (12)	0.0448 (4)	
H16	0.348824	0.682066	0.970723	0.054*	
C17	0.16167 (15)	0.27789 (11)	0.88911 (10)	0.0382 (3)	
C18	0.01724 (19)	0.20457 (18)	0.77497 (14)	0.0667 (6)	
H18A	0.013683	0.185606	0.715136	0.080*	
H18B	−0.025021	0.262259	0.772271	0.080*	
C19	−0.0413 (2)	0.13481 (18)	0.8174 (2)	0.0801 (8)	
H19A	−0.122184	0.127782	0.784298	0.120*	
H19B	−0.000616	0.077309	0.819137	0.120*	
H19C	−0.039294	0.153913	0.876235	0.120*	
C20	0.37399 (16)	0.21008 (12)	0.92377 (11)	0.0431 (4)	
H20A	0.353151	0.183237	0.865339	0.052*	
H20B	0.455297	0.231203	0.934593	0.052*	
C21	0.37065 (15)	0.13484 (11)	0.98875 (11)	0.0415 (3)	
C22	0.27325 (18)	0.07926 (13)	0.98202 (13)	0.0523 (4)	
H22	0.204724	0.090984	0.938080	0.063*	
C23	0.2745 (2)	0.00668 (13)	1.03865 (14)	0.0571 (5)	
H23	0.207476	−0.029598	1.033520	0.069*	
C24	0.3756 (2)	−0.01102 (13)	1.10227 (14)	0.0547 (5)	
C25	0.4723 (2)	0.04360 (18)	1.11285 (17)	0.0731 (7)	
H25	0.539652	0.032188	1.157984	0.088*	
C26	0.47006 (18)	0.11658 (16)	1.05584 (16)	0.0635 (6)	
H26	0.536548	0.153845	1.062887	0.076*	

### Diethyl 1-(4-chlorobenzyl)-4-(4-chlorophenyl)-2,2-dioxo-3,4,6,7,8,8a-hexahydro-1H-pyrrolo[2,1-c][1,4]thiazine-1,3-dicarboxylate (I) Atomic displacement parameters (Å^2^)

**Table:**

	*U*^11^	*U*^22^	*U*^33^	*U*^12^	*U*^13^	*U*^23^
S1	0.03340 (18)	0.03586 (19)	0.02935 (16)	0.00057 (14)	0.00751 (13)	−0.00005 (13)
Cl1	0.0653 (3)	0.0548 (3)	0.0799 (4)	0.0222 (2)	0.0206 (3)	0.0083 (2)
Cl2	0.1097 (5)	0.0584 (4)	0.0927 (4)	0.0177 (3)	0.0348 (4)	0.0280 (3)
O1	0.0329 (6)	0.0545 (7)	0.0421 (6)	0.0022 (5)	0.0037 (4)	−0.0009 (5)
O2	0.0529 (7)	0.0419 (6)	0.0376 (5)	0.0011 (5)	0.0168 (5)	0.0049 (5)
O3	0.0503 (8)	0.0792 (10)	0.0645 (9)	−0.0028 (7)	−0.0005 (7)	−0.0298 (8)
O4	0.0737 (9)	0.0586 (8)	0.0534 (7)	−0.0123 (7)	0.0323 (7)	−0.0186 (6)
O5	0.0390 (6)	0.0577 (8)	0.0591 (7)	−0.0067 (5)	0.0149 (5)	−0.0205 (6)
O6	0.0480 (7)	0.0621 (8)	0.0435 (6)	0.0037 (6)	0.0003 (5)	−0.0200 (6)
N1	0.0392 (7)	0.0397 (7)	0.0301 (6)	0.0000 (5)	0.0095 (5)	0.0021 (5)
C1	0.0376 (7)	0.0366 (7)	0.0306 (6)	0.0006 (6)	0.0113 (6)	−0.0020 (5)
C2	0.0431 (8)	0.0424 (8)	0.0349 (7)	0.0000 (7)	0.0162 (6)	0.0007 (6)
C3	0.1002 (17)	0.0582 (12)	0.0410 (9)	−0.0006 (11)	0.0350 (10)	−0.0019 (8)
C4	0.0897 (17)	0.0819 (16)	0.0406 (9)	0.0261 (13)	0.0251 (10)	0.0129 (10)
C5	0.0609 (11)	0.0547 (11)	0.0379 (8)	−0.0010 (9)	0.0148 (8)	0.0113 (7)
C6	0.0314 (7)	0.0361 (7)	0.0342 (7)	−0.0021 (6)	0.0058 (5)	0.0017 (6)
C7	0.0333 (7)	0.0348 (7)	0.0312 (6)	0.0005 (6)	0.0065 (5)	−0.0028 (5)
C8	0.0484 (9)	0.0382 (8)	0.0346 (7)	0.0022 (7)	0.0064 (7)	−0.0016 (6)
C9	0.114 (2)	0.0633 (13)	0.0550 (11)	−0.0095 (13)	0.0410 (12)	−0.0196 (10)
C10	0.107 (2)	0.0549 (13)	0.0810 (16)	0.0131 (13)	0.0248 (15)	−0.0163 (12)
C11	0.0346 (7)	0.0370 (8)	0.0362 (7)	0.0006 (6)	0.0074 (6)	0.0043 (6)
C12	0.0371 (8)	0.0443 (9)	0.0542 (10)	−0.0027 (7)	0.0034 (7)	−0.0002 (7)
C13	0.0358 (8)	0.0561 (11)	0.0536 (10)	0.0043 (7)	0.0059 (7)	0.0047 (8)
C14	0.0475 (9)	0.0433 (9)	0.0426 (8)	0.0104 (7)	0.0141 (7)	0.0071 (7)
C15	0.0502 (10)	0.0360 (9)	0.0607 (11)	−0.0019 (7)	0.0121 (8)	0.0010 (7)
C16	0.0361 (8)	0.0408 (9)	0.0552 (10)	−0.0026 (7)	0.0074 (7)	0.0026 (7)
C17	0.0413 (8)	0.0383 (8)	0.0340 (7)	−0.0017 (6)	0.0075 (6)	−0.0022 (6)
C18	0.0517 (11)	0.0873 (16)	0.0486 (10)	0.0048 (11)	−0.0106 (9)	−0.0256 (11)
C19	0.0570 (13)	0.0634 (14)	0.107 (2)	−0.0067 (11)	−0.0032 (13)	−0.0201 (14)
C20	0.0455 (9)	0.0421 (9)	0.0447 (8)	0.0061 (7)	0.0168 (7)	−0.0029 (7)
C21	0.0433 (9)	0.0363 (8)	0.0461 (8)	0.0057 (6)	0.0132 (7)	−0.0055 (6)
C22	0.0509 (10)	0.0435 (9)	0.0558 (10)	−0.0004 (8)	0.0010 (8)	−0.0016 (8)
C23	0.0606 (12)	0.0406 (10)	0.0678 (12)	−0.0057 (8)	0.0119 (10)	−0.0026 (8)
C24	0.0657 (12)	0.0417 (10)	0.0597 (11)	0.0113 (9)	0.0216 (9)	0.0065 (8)
C25	0.0526 (12)	0.0780 (16)	0.0804 (15)	0.0089 (11)	0.0011 (11)	0.0285 (13)
C26	0.0415 (10)	0.0639 (13)	0.0795 (14)	−0.0003 (9)	0.0047 (9)	0.0156 (11)

### Diethyl 1-(4-chlorobenzyl)-4-(4-chlorophenyl)-2,2-dioxo-3,4,6,7,8,8a-hexahydro-1H-pyrrolo[2,1-c][1,4]thiazine-1,3-dicarboxylate (I) Geometric parameters (Å, º)

**Table:**

S1—O2	1.4303 (12)	C9—H9A	0.9700
S1—O1	1.4352 (12)	C9—H9B	0.9700
S1—C7	1.7921 (15)	C10—H10A	0.9600
S1—C1	1.8181 (14)	C10—H10B	0.9600
Cl1—C14	1.7375 (17)	C10—H10C	0.9600
Cl2—C24	1.738 (2)	C11—C16	1.379 (2)
O3—C8	1.183 (2)	C11—C12	1.389 (2)
O4—C8	1.319 (2)	C12—C13	1.375 (3)
O4—C9	1.461 (2)	C12—H12	0.9300
O5—C17	1.186 (2)	C13—C14	1.380 (3)
O6—C17	1.3326 (19)	C13—H13	0.9300
O6—C18	1.463 (2)	C14—C15	1.371 (3)
N1—C6	1.4623 (19)	C15—C16	1.379 (3)
N1—C2	1.465 (2)	C15—H15	0.9300
N1—C5	1.476 (2)	C16—H16	0.9300
C1—C17	1.524 (2)	C18—C19	1.474 (4)
C1—C20	1.548 (2)	C18—H18A	0.9700
C1—C2	1.552 (2)	C18—H18B	0.9700
C2—C3	1.525 (2)	C19—H19A	0.9600
C2—H2	0.9800	C19—H19B	0.9600
C3—C4	1.487 (3)	C19—H19C	0.9600
C3—H3A	0.9700	C20—C21	1.505 (2)
C3—H3B	0.9700	C20—H20A	0.9700
C4—C5	1.502 (3)	C20—H20B	0.9700
C4—H4A	0.9700	C21—C22	1.377 (3)
C4—H4B	0.9700	C21—C26	1.381 (3)
C5—H5A	0.9700	C22—C23	1.380 (3)
C5—H5B	0.9700	C22—H22	0.9300
C6—C11	1.515 (2)	C23—C24	1.363 (3)
C6—C7	1.542 (2)	C23—H23	0.9300
C6—H6	0.9800	C24—C25	1.356 (3)
C7—C8	1.524 (2)	C25—C26	1.386 (3)
C7—H7	0.9800	C25—H25	0.9300
C9—C10	1.469 (4)	C26—H26	0.9300
			
O2—S1—O1	118.25 (7)	C9—C10—H10B	109.5
O2—S1—C7	107.77 (7)	H10A—C10—H10B	109.5
O1—S1—C7	109.55 (7)	C9—C10—H10C	109.5
O2—S1—C1	112.24 (7)	H10A—C10—H10C	109.5
O1—S1—C1	105.24 (7)	H10B—C10—H10C	109.5
C7—S1—C1	102.68 (7)	C16—C11—C12	118.66 (15)
C8—O4—C9	115.89 (17)	C16—C11—C6	120.26 (14)
C17—O6—C18	116.64 (14)	C12—C11—C6	120.98 (14)
C6—N1—C2	113.12 (12)	C13—C12—C11	121.09 (17)
C6—N1—C5	111.57 (13)	C13—C12—H12	119.5
C2—N1—C5	104.44 (13)	C11—C12—H12	119.5
C17—C1—C20	115.13 (13)	C12—C13—C14	118.91 (16)
C17—C1—C2	110.24 (12)	C12—C13—H13	120.5
C20—C1—C2	108.55 (12)	C14—C13—H13	120.5
C17—C1—S1	110.17 (10)	C15—C14—C13	121.04 (16)
C20—C1—S1	107.57 (10)	C15—C14—Cl1	119.66 (14)
C2—C1—S1	104.61 (10)	C13—C14—Cl1	119.30 (14)
N1—C2—C3	104.15 (14)	C14—C15—C16	119.46 (17)
N1—C2—C1	111.90 (12)	C14—C15—H15	120.3
C3—C2—C1	115.67 (14)	C16—C15—H15	120.3
N1—C2—H2	108.3	C15—C16—C11	120.84 (16)
C3—C2—H2	108.3	C15—C16—H16	119.6
C1—C2—H2	108.3	C11—C16—H16	119.6
C4—C3—C2	104.70 (16)	O5—C17—O6	124.55 (15)
C4—C3—H3A	110.8	O5—C17—C1	125.33 (14)
C2—C3—H3A	110.8	O6—C17—C1	110.05 (13)
C4—C3—H3B	110.8	O6—C18—C19	111.90 (19)
C2—C3—H3B	110.8	O6—C18—H18A	109.2
H3A—C3—H3B	108.9	C19—C18—H18A	109.2
C3—C4—C5	107.14 (15)	O6—C18—H18B	109.2
C3—C4—H4A	110.3	C19—C18—H18B	109.2
C5—C4—H4A	110.3	H18A—C18—H18B	107.9
C3—C4—H4B	110.3	C18—C19—H19A	109.5
C5—C4—H4B	110.3	C18—C19—H19B	109.5
H4A—C4—H4B	108.5	H19A—C19—H19B	109.5
N1—C5—C4	105.07 (16)	C18—C19—H19C	109.5
N1—C5—H5A	110.7	H19A—C19—H19C	109.5
C4—C5—H5A	110.7	H19B—C19—H19C	109.5
N1—C5—H5B	110.7	C21—C20—C1	118.45 (13)
C4—C5—H5B	110.7	C21—C20—H20A	107.7
H5A—C5—H5B	108.8	C1—C20—H20A	107.7
N1—C6—C11	111.45 (12)	C21—C20—H20B	107.7
N1—C6—C7	110.73 (12)	C1—C20—H20B	107.7
C11—C6—C7	107.06 (12)	H20A—C20—H20B	107.1
N1—C6—H6	109.2	C22—C21—C26	117.53 (18)
C11—C6—H6	109.2	C22—C21—C20	122.59 (16)
C7—C6—H6	109.2	C26—C21—C20	119.80 (17)
C8—C7—C6	109.74 (12)	C21—C22—C23	121.82 (18)
C8—C7—S1	107.75 (10)	C21—C22—H22	119.1
C6—C7—S1	113.64 (10)	C23—C22—H22	119.1
C8—C7—H7	108.5	C24—C23—C22	118.9 (2)
C6—C7—H7	108.5	C24—C23—H23	120.5
S1—C7—H7	108.5	C22—C23—H23	120.5
O3—C8—O4	125.41 (16)	C25—C24—C23	121.14 (19)
O3—C8—C7	123.87 (16)	C25—C24—Cl2	119.31 (17)
O4—C8—C7	110.70 (14)	C23—C24—Cl2	119.54 (17)
O4—C9—C10	112.64 (19)	C24—C25—C26	119.5 (2)
O4—C9—H9A	109.1	C24—C25—H25	120.3
C10—C9—H9A	109.1	C26—C25—H25	120.3
O4—C9—H9B	109.1	C21—C26—C25	121.1 (2)
C10—C9—H9B	109.1	C21—C26—H26	119.5
H9A—C9—H9B	107.8	C25—C26—H26	119.5
C9—C10—H10A	109.5		
			
O2—S1—C1—C17	48.56 (13)	S1—C7—C8—O3	66.9 (2)
O1—S1—C1—C17	178.45 (11)	C6—C7—C8—O4	121.01 (15)
C7—S1—C1—C17	−66.91 (12)	S1—C7—C8—O4	−114.79 (13)
O2—S1—C1—C20	−77.67 (12)	C8—O4—C9—C10	72.6 (3)
O1—S1—C1—C20	52.23 (12)	N1—C6—C11—C16	−127.69 (16)
C7—S1—C1—C20	166.86 (11)	C7—C6—C11—C16	111.09 (16)
O2—S1—C1—C2	167.03 (10)	N1—C6—C11—C12	55.95 (19)
O1—S1—C1—C2	−63.07 (11)	C7—C6—C11—C12	−65.28 (18)
C7—S1—C1—C2	51.56 (11)	C16—C11—C12—C13	−0.4 (3)
C6—N1—C2—C3	−159.98 (14)	C6—C11—C12—C13	176.01 (16)
C5—N1—C2—C3	−38.47 (17)	C11—C12—C13—C14	−0.3 (3)
C6—N1—C2—C1	74.37 (16)	C12—C13—C14—C15	0.9 (3)
C5—N1—C2—C1	−164.12 (13)	C12—C13—C14—Cl1	−179.04 (15)
C17—C1—C2—N1	51.72 (16)	C13—C14—C15—C16	−0.7 (3)
C20—C1—C2—N1	178.67 (12)	Cl1—C14—C15—C16	179.20 (15)
S1—C1—C2—N1	−66.71 (14)	C14—C15—C16—C11	0.0 (3)
C17—C1—C2—C3	−67.33 (19)	C12—C11—C16—C15	0.6 (3)
C20—C1—C2—C3	59.6 (2)	C6—C11—C16—C15	−175.87 (16)
S1—C1—C2—C3	174.25 (14)	C18—O6—C17—O5	5.9 (3)
N1—C2—C3—C4	28.0 (2)	C18—O6—C17—C1	−171.25 (16)
C1—C2—C3—C4	151.21 (18)	C20—C1—C17—O5	144.79 (17)
C2—C3—C4—C5	−7.1 (3)	C2—C1—C17—O5	−92.02 (19)
C6—N1—C5—C4	156.59 (15)	S1—C1—C17—O5	22.9 (2)
C2—N1—C5—C4	34.06 (19)	C20—C1—C17—O6	−38.06 (18)
C3—C4—C5—N1	−16.2 (2)	C2—C1—C17—O6	85.14 (16)
C2—N1—C6—C11	177.61 (12)	S1—C1—C17—O6	−159.90 (12)
C5—N1—C6—C11	60.20 (17)	C17—O6—C18—C19	−86.0 (2)
C2—N1—C6—C7	−63.33 (16)	C17—C1—C20—C21	−68.67 (19)
C5—N1—C6—C7	179.26 (13)	C2—C1—C20—C21	167.23 (14)
N1—C6—C7—C8	173.85 (12)	S1—C1—C20—C21	54.56 (18)
C11—C6—C7—C8	−64.47 (15)	C1—C20—C21—C22	69.0 (2)
N1—C6—C7—S1	53.15 (14)	C1—C20—C21—C26	−114.3 (2)
C11—C6—C7—S1	174.82 (10)	C26—C21—C22—C23	−1.3 (3)
O2—S1—C7—C8	71.37 (12)	C20—C21—C22—C23	175.54 (18)
O1—S1—C7—C8	−58.51 (12)	C21—C22—C23—C24	−0.8 (3)
C1—S1—C7—C8	−169.97 (11)	C22—C23—C24—C25	2.8 (3)
O2—S1—C7—C6	−166.81 (10)	C22—C23—C24—Cl2	−176.68 (16)
O1—S1—C7—C6	63.30 (12)	C23—C24—C25—C26	−2.6 (4)
C1—S1—C7—C6	−48.16 (12)	Cl2—C24—C25—C26	176.9 (2)
C9—O4—C8—O3	3.5 (3)	C22—C21—C26—C25	1.5 (3)
C9—O4—C8—C7	−174.77 (16)	C20—C21—C26—C25	−175.4 (2)
C6—C7—C8—O3	−57.3 (2)	C24—C25—C26—C21	0.4 (4)

### Diethyl 1-(4-chlorobenzyl)-4-(4-chlorophenyl)-2,2-dioxo-3,4,6,7,8,8a-hexahydro-1H-pyrrolo[2,1-c][1,4]thiazine-1,3-dicarboxylate (I) Hydrogen-bond geometry (Å, º)

**Table:**

*D*—H···*A*	*D*—H	H···*A*	*D*···*A*	*D*—H···*A*
C16—H16···O1^i^	0.93	2.50	3.335 (2)	149
C18—H18*A*···O1^ii^	0.97	2.45	3.397 (2)	166

Symmetry codes: (i) −*x*+1, −*y*+1, −*z*+2; (ii) *x*−1/2, −*y*+1/2, *z*−1/2.

### Diethyl 1-(4-methylbenzyl)-4-(4-methylphenyl)-2,2-dioxo-3,4,6,7,8,8a-hexahydro-1H-pyrrolo[2,1-c][1,4]thiazine-1,3-dicarboxylate (II) Crystal data

**Table:**

C_28_H_35_NO_6_S	*F*(000) = 1096
*M**_r_* = 513.63	*D*_x_ = 1.296 Mg m^−^^3^*D*_m_ = 1.29 Mg m^−^^3^*D*_m_ measured by floatation method
Monoclinic, *P*2_1_/*n*	Mo *K*α radiation, λ = 0.71073 Å
*a* = 11.8641 (5) Å	Cell parameters from 5161 reflections
*b* = 14.4765 (6) Å	θ = 4.8–59.1°
*c* = 15.8654 (7) Å	µ = 0.17 mm^−^^1^
β = 104.960 (2)°	*T* = 293 K
*V* = 2632.5 (2) Å^3^	Block, colorless
*Z* = 4	0.26 × 0.22 × 0.20 mm

### Diethyl 1-(4-methylbenzyl)-4-(4-methylphenyl)-2,2-dioxo-3,4,6,7,8,8a-hexahydro-1H-pyrrolo[2,1-c][1,4]thiazine-1,3-dicarboxylate (II) Data collection

**Table:**

Bruker SMART APEXII CCD diffractometer	5294 reflections with *I* > 2σ(*I*)
Radiation source: fine-focus sealed tube	*R*_int_ = 0.032
φ and ω scans	θ_max_ = 30.5°, θ_min_ = 1.9°
Absorption correction: multi-scan (*SADABS*; Bruker, 2009)	*h* = −16→16
*T*_min_ = 0.863, *T*_max_ = 1.000	*k* = −20→19
34960 measured reflections	*l* = −22→19
7985 independent reflections	

### Diethyl 1-(4-methylbenzyl)-4-(4-methylphenyl)-2,2-dioxo-3,4,6,7,8,8a-hexahydro-1H-pyrrolo[2,1-c][1,4]thiazine-1,3-dicarboxylate (II) Refinement

**Table:**

Refinement on *F*^2^	0 restraints
Least-squares matrix: full	Hydrogen site location: inferred from neighbouring sites
*R*[*F*^2^ > 2σ(*F*^2^)] = 0.051	H-atom parameters constrained
*wR*(*F*^2^) = 0.163	*w* = 1/[σ^2^(*F*_o_^2^) + (0.0833*P*)^2^ + 0.5606*P*] where *P* = (*F*_o_^2^ + 2*F*_c_^2^)/3
*S* = 1.03	(Δ/σ)_max_ = 0.002
7985 reflections	Δρ_max_ = 0.36 e Å^−^^3^
325 parameters	Δρ_min_ = −0.22 e Å^−^^3^

### Diethyl 1-(4-methylbenzyl)-4-(4-methylphenyl)-2,2-dioxo-3,4,6,7,8,8a-hexahydro-1H-pyrrolo[2,1-c][1,4]thiazine-1,3-dicarboxylate (II) Special details

**Table:**

Geometry. All esds (except the esd in the dihedral angle between two l.s. planes) are estimated using the full covariance matrix. The cell esds are taken into account individually in the estimation of esds in distances, angles and torsion angles; correlations between esds in cell parameters are only used when they are defined by crystal symmetry. An approximate (isotropic) treatment of cell esds is used for estimating esds involving l.s. planes.

### Diethyl 1-(4-methylbenzyl)-4-(4-methylphenyl)-2,2-dioxo-3,4,6,7,8,8a-hexahydro-1H-pyrrolo[2,1-c][1,4]thiazine-1,3-dicarboxylate (II) Fractional atomic coordinates and isotropic or equivalent isotropic displacement parameters (Å^2^)

**Table:**

	*x*	*y*	*z*	*U*_iso_*/*U*_eq_	
S1	0.33590 (3)	0.66176 (3)	1.04119 (2)	0.03672 (12)	
O1	0.45970 (10)	0.64748 (9)	1.06758 (8)	0.0481 (3)	
O2	0.28369 (11)	0.71408 (8)	1.09751 (8)	0.0470 (3)	
O3	0.39277 (12)	0.46595 (12)	1.13588 (10)	0.0692 (4)	
O4	0.20861 (13)	0.48525 (10)	1.13971 (9)	0.0614 (4)	
O5	0.10131 (11)	0.69708 (10)	0.92535 (9)	0.0544 (3)	
O6	0.15751 (12)	0.78342 (10)	0.82750 (8)	0.0569 (4)	
N1	0.26795 (12)	0.55299 (10)	0.86792 (8)	0.0389 (3)	
C1	0.30621 (14)	0.71013 (11)	0.93172 (10)	0.0375 (3)	
C2	0.34466 (15)	0.63369 (12)	0.87672 (11)	0.0414 (4)	
H2	0.423774	0.614248	0.907295	0.050*	
C3	0.3446 (2)	0.65906 (15)	0.78307 (13)	0.0601 (5)	
H3A	0.294614	0.711960	0.763165	0.072*	
H3B	0.422883	0.673687	0.779229	0.072*	
C4	0.2993 (3)	0.57610 (18)	0.73004 (14)	0.0774 (7)	
H4A	0.352341	0.557578	0.695710	0.093*	
H4B	0.223547	0.588810	0.690802	0.093*	
C5	0.28986 (19)	0.50135 (15)	0.79370 (12)	0.0536 (5)	
H5A	0.225872	0.459708	0.768792	0.064*	
H5B	0.361618	0.466066	0.811293	0.064*	
C6	0.28984 (13)	0.49651 (11)	0.94697 (10)	0.0374 (3)	
H6	0.371873	0.477439	0.963365	0.045*	
C7	0.26332 (13)	0.55239 (11)	1.02239 (10)	0.0360 (3)	
H7	0.179024	0.563348	1.008656	0.043*	
C8	0.29827 (15)	0.49644 (12)	1.10607 (11)	0.0422 (4)	
C9	0.2268 (3)	0.42380 (17)	1.21509 (16)	0.0785 (7)	
H9A	0.165274	0.433703	1.244232	0.094*	
H9B	0.300563	0.438652	1.256026	0.094*	
C10	0.2273 (3)	0.32666 (18)	1.1897 (2)	0.0920 (9)	
H10A	0.239383	0.288404	1.240731	0.138*	
H10B	0.153808	0.311446	1.150054	0.138*	
H10C	0.288994	0.316392	1.161839	0.138*	
C11	0.21321 (14)	0.41175 (12)	0.93370 (11)	0.0390 (3)	
C12	0.09207 (15)	0.41939 (13)	0.91062 (13)	0.0486 (4)	
H12	0.057668	0.477442	0.900477	0.058*	
C13	0.02312 (16)	0.34248 (14)	0.90269 (13)	0.0505 (4)	
H13	−0.057565	0.349263	0.887189	0.061*	
C14	0.07066 (16)	0.25490 (13)	0.91721 (12)	0.0470 (4)	
C14A	−0.0061 (2)	0.17109 (16)	0.90913 (17)	0.0671 (6)	
H14A	−0.086463	0.189820	0.893354	0.101*	
H14B	0.008329	0.130876	0.864951	0.101*	
H14C	0.010726	0.139017	0.963930	0.101*	
C15	0.19089 (17)	0.24754 (13)	0.93940 (13)	0.0507 (4)	
H15	0.225235	0.189437	0.949129	0.061*	
C16	0.26104 (15)	0.32506 (12)	0.94740 (13)	0.0463 (4)	
H16	0.341720	0.318298	0.962260	0.056*	
C17	0.17625 (15)	0.72887 (12)	0.89707 (11)	0.0402 (4)	
C18	0.03628 (19)	0.79511 (19)	0.77812 (14)	0.0709 (7)	
H18A	−0.004747	0.737054	0.777501	0.085*	
H18B	0.033985	0.810862	0.718277	0.085*	
C19	−0.0236 (2)	0.86713 (19)	0.8148 (2)	0.0895 (9)	
H19A	−0.102775	0.872332	0.780277	0.134*	
H19B	−0.023044	0.851248	0.873615	0.134*	
H19C	0.015681	0.925041	0.814403	0.134*	
C20	0.38532 (16)	0.79579 (13)	0.93554 (12)	0.0463 (4)	
H20A	0.465523	0.774455	0.949683	0.056*	
H20B	0.368509	0.822013	0.877356	0.056*	
C21	0.37805 (16)	0.87256 (12)	0.99772 (12)	0.0452 (4)	
C22	0.2812 (2)	0.92793 (15)	0.98748 (15)	0.0625 (5)	
H22	0.215341	0.915392	0.942459	0.075*	
C23	0.2791 (2)	1.00134 (15)	1.04211 (17)	0.0671 (6)	
H23	0.211776	1.036793	1.033849	0.080*	
C24	0.3747 (2)	1.02312 (15)	1.10852 (15)	0.0600 (5)	
C24A	0.3743 (3)	1.10687 (18)	1.1650 (2)	0.0882 (8)	
H24A	0.299692	1.136819	1.147269	0.132*	
H24B	0.388762	1.088117	1.224862	0.132*	
H24C	0.434114	1.148974	1.158740	0.132*	
C25	0.4700 (2)	0.96798 (19)	1.11975 (17)	0.0748 (7)	
H25	0.535644	0.980826	1.164838	0.090*	
C26	0.47207 (18)	0.89356 (17)	1.06612 (16)	0.0661 (6)	
H26	0.538450	0.856724	1.076391	0.079*	

### Diethyl 1-(4-methylbenzyl)-4-(4-methylphenyl)-2,2-dioxo-3,4,6,7,8,8a-hexahydro-1H-pyrrolo[2,1-c][1,4]thiazine-1,3-dicarboxylate (II) Atomic displacement parameters (Å^2^)

**Table:**

	*U*^11^	*U*^22^	*U*^33^	*U*^12^	*U*^13^	*U*^23^
S1	0.0372 (2)	0.0420 (2)	0.0315 (2)	0.00073 (16)	0.00974 (15)	0.00117 (16)
O1	0.0367 (6)	0.0609 (8)	0.0442 (7)	−0.0017 (5)	0.0060 (5)	0.0021 (6)
O2	0.0586 (7)	0.0470 (7)	0.0399 (6)	0.0019 (6)	0.0207 (6)	−0.0029 (5)
O3	0.0511 (8)	0.0829 (11)	0.0668 (9)	0.0067 (7)	0.0029 (7)	0.0315 (8)
O4	0.0788 (10)	0.0605 (8)	0.0567 (8)	0.0195 (7)	0.0390 (7)	0.0217 (7)
O5	0.0416 (7)	0.0645 (8)	0.0600 (8)	0.0097 (6)	0.0181 (6)	0.0242 (7)
O6	0.0516 (7)	0.0703 (9)	0.0440 (7)	−0.0039 (6)	0.0034 (6)	0.0224 (7)
N1	0.0397 (7)	0.0451 (8)	0.0331 (7)	0.0017 (6)	0.0115 (5)	−0.0002 (6)
C1	0.0382 (8)	0.0418 (9)	0.0347 (8)	−0.0003 (7)	0.0132 (6)	0.0038 (7)
C2	0.0437 (8)	0.0476 (9)	0.0370 (8)	−0.0004 (7)	0.0179 (7)	0.0004 (7)
C3	0.0853 (15)	0.0619 (12)	0.0424 (10)	−0.0003 (11)	0.0333 (10)	0.0024 (9)
C4	0.1073 (19)	0.0896 (17)	0.0390 (10)	−0.0316 (15)	0.0258 (11)	−0.0069 (11)
C5	0.0622 (11)	0.0620 (12)	0.0393 (9)	−0.0029 (9)	0.0180 (8)	−0.0099 (8)
C6	0.0336 (7)	0.0414 (8)	0.0369 (8)	0.0031 (6)	0.0085 (6)	0.0002 (7)
C7	0.0356 (7)	0.0395 (8)	0.0329 (7)	0.0028 (6)	0.0087 (6)	0.0048 (6)
C8	0.0478 (9)	0.0408 (9)	0.0360 (8)	0.0017 (7)	0.0071 (7)	0.0025 (7)
C9	0.121 (2)	0.0677 (14)	0.0610 (13)	0.0211 (14)	0.0495 (14)	0.0257 (12)
C10	0.121 (2)	0.0649 (16)	0.091 (2)	−0.0210 (15)	0.0281 (18)	0.0176 (14)
C11	0.0367 (8)	0.0443 (9)	0.0362 (8)	0.0014 (7)	0.0100 (6)	−0.0013 (7)
C12	0.0389 (9)	0.0482 (10)	0.0560 (11)	0.0053 (7)	0.0072 (7)	0.0027 (9)
C13	0.0359 (8)	0.0604 (12)	0.0539 (11)	−0.0007 (8)	0.0092 (7)	−0.0024 (9)
C14	0.0499 (9)	0.0504 (10)	0.0427 (9)	−0.0069 (8)	0.0154 (8)	−0.0078 (8)
C14A	0.0636 (13)	0.0617 (13)	0.0794 (15)	−0.0170 (10)	0.0246 (12)	−0.0110 (11)
C15	0.0523 (10)	0.0405 (9)	0.0595 (11)	0.0019 (8)	0.0151 (9)	−0.0057 (8)
C16	0.0378 (8)	0.0462 (10)	0.0553 (10)	0.0040 (7)	0.0125 (7)	−0.0035 (8)
C17	0.0438 (8)	0.0401 (8)	0.0367 (8)	0.0027 (7)	0.0104 (7)	0.0042 (7)
C18	0.0547 (12)	0.0952 (18)	0.0518 (12)	−0.0029 (12)	−0.0064 (9)	0.0278 (12)
C19	0.0639 (15)	0.0751 (17)	0.114 (2)	0.0094 (13)	−0.0057 (14)	0.0259 (16)
C20	0.0463 (9)	0.0505 (10)	0.0460 (9)	−0.0052 (8)	0.0189 (7)	0.0042 (8)
C21	0.0478 (9)	0.0427 (9)	0.0474 (10)	−0.0062 (8)	0.0166 (8)	0.0055 (8)
C22	0.0625 (12)	0.0504 (11)	0.0657 (13)	0.0049 (9)	0.0007 (10)	−0.0013 (10)
C23	0.0714 (14)	0.0505 (12)	0.0778 (15)	0.0126 (10)	0.0165 (12)	0.0022 (11)
C24	0.0737 (14)	0.0489 (11)	0.0644 (13)	−0.0112 (10)	0.0304 (11)	−0.0026 (10)
C24A	0.116 (2)	0.0635 (15)	0.096 (2)	−0.0161 (15)	0.0468 (17)	−0.0197 (14)
C25	0.0598 (13)	0.0840 (17)	0.0761 (16)	−0.0124 (12)	0.0094 (11)	−0.0278 (14)
C26	0.0445 (10)	0.0749 (15)	0.0757 (15)	−0.0011 (10)	0.0098 (10)	−0.0146 (12)

### Diethyl 1-(4-methylbenzyl)-4-(4-methylphenyl)-2,2-dioxo-3,4,6,7,8,8a-hexahydro-1H-pyrrolo[2,1-c][1,4]thiazine-1,3-dicarboxylate (II) Geometric parameters (Å, º)

**Table:**

S1—O2	1.4287 (12)	C11—C16	1.371 (2)
S1—O1	1.4345 (12)	C11—C12	1.393 (2)
S1—C7	1.7901 (17)	C12—C13	1.368 (3)
S1—C1	1.8208 (16)	C12—H12	0.9300
O3—C8	1.184 (2)	C13—C14	1.383 (3)
O4—C8	1.317 (2)	C13—H13	0.9300
O4—C9	1.461 (2)	C14—C15	1.382 (3)
O5—C17	1.187 (2)	C14—C14A	1.503 (3)
O6—C17	1.328 (2)	C14A—H14A	0.9600
O6—C18	1.459 (2)	C14A—H14B	0.9600
N1—C6	1.463 (2)	C14A—H14C	0.9600
N1—C2	1.465 (2)	C15—C16	1.383 (3)
N1—C5	1.474 (2)	C15—H15	0.9300
C1—C17	1.522 (2)	C16—H16	0.9300
C1—C20	1.547 (2)	C18—C19	1.464 (4)
C1—C2	1.549 (2)	C18—H18A	0.9700
C2—C3	1.530 (2)	C18—H18B	0.9700
C2—H2	0.9800	C19—H19A	0.9600
C3—C4	1.485 (3)	C19—H19B	0.9600
C3—H3A	0.9700	C19—H19C	0.9600
C3—H3B	0.9700	C20—C21	1.503 (3)
C4—C5	1.504 (3)	C20—H20A	0.9700
C4—H4A	0.9700	C20—H20B	0.9700
C4—H4B	0.9700	C21—C26	1.374 (3)
C5—H5A	0.9700	C21—C22	1.376 (3)
C5—H5B	0.9700	C22—C23	1.375 (3)
C6—C11	1.509 (2)	C22—H22	0.9300
C6—C7	1.543 (2)	C23—C24	1.370 (3)
C6—H6	0.9800	C23—H23	0.9300
C7—C8	1.519 (2)	C24—C25	1.358 (3)
C7—H7	0.9800	C24—C24A	1.508 (3)
C9—C10	1.463 (4)	C24A—H24A	0.9600
C9—H9A	0.9700	C24A—H24B	0.9600
C9—H9B	0.9700	C24A—H24C	0.9600
C10—H10A	0.9600	C25—C26	1.377 (3)
C10—H10B	0.9600	C25—H25	0.9300
C10—H10C	0.9600	C26—H26	0.9300
			
O2—S1—O1	118.06 (8)	C16—C11—C6	120.81 (15)
O2—S1—C7	107.80 (8)	C12—C11—C6	121.04 (15)
O1—S1—C7	109.48 (8)	C13—C12—C11	120.77 (17)
O2—S1—C1	112.60 (8)	C13—C12—H12	119.6
O1—S1—C1	105.37 (7)	C11—C12—H12	119.6
C7—S1—C1	102.41 (7)	C12—C13—C14	121.50 (17)
C8—O4—C9	116.26 (17)	C12—C13—H13	119.3
C17—O6—C18	116.44 (15)	C14—C13—H13	119.3
C6—N1—C2	113.30 (13)	C15—C14—C13	117.57 (17)
C6—N1—C5	111.88 (14)	C15—C14—C14A	121.49 (19)
C2—N1—C5	104.32 (13)	C13—C14—C14A	120.93 (18)
C17—C1—C20	114.78 (14)	C14—C14A—H14A	109.5
C17—C1—C2	110.01 (13)	C14—C14A—H14B	109.5
C20—C1—C2	109.32 (13)	H14A—C14A—H14B	109.5
C17—C1—S1	109.98 (11)	C14—C14A—H14C	109.5
C20—C1—S1	107.75 (11)	H14A—C14A—H14C	109.5
C2—C1—S1	104.48 (11)	H14B—C14A—H14C	109.5
N1—C2—C3	104.72 (15)	C14—C15—C16	121.18 (17)
N1—C2—C1	110.93 (12)	C14—C15—H15	119.4
C3—C2—C1	116.68 (15)	C16—C15—H15	119.4
N1—C2—H2	108.1	C11—C16—C15	120.89 (16)
C3—C2—H2	108.1	C11—C16—H16	119.6
C1—C2—H2	108.1	C15—C16—H16	119.6
C4—C3—C2	105.40 (16)	O5—C17—O6	124.30 (16)
C4—C3—H3A	110.7	O5—C17—C1	125.60 (15)
C2—C3—H3A	110.7	O6—C17—C1	110.05 (14)
C4—C3—H3B	110.7	O6—C18—C19	112.4 (2)
C2—C3—H3B	110.7	O6—C18—H18A	109.1
H3A—C3—H3B	108.8	C19—C18—H18A	109.1
C3—C4—C5	106.27 (17)	O6—C18—H18B	109.1
C3—C4—H4A	110.5	C19—C18—H18B	109.1
C5—C4—H4A	110.5	H18A—C18—H18B	107.8
C3—C4—H4B	110.5	C18—C19—H19A	109.5
C5—C4—H4B	110.5	C18—C19—H19B	109.5
H4A—C4—H4B	108.7	H19A—C19—H19B	109.5
N1—C5—C4	103.34 (17)	C18—C19—H19C	109.5
N1—C5—H5A	111.1	H19A—C19—H19C	109.5
C4—C5—H5A	111.1	H19B—C19—H19C	109.5
N1—C5—H5B	111.1	C21—C20—C1	118.77 (14)
C4—C5—H5B	111.1	C21—C20—H20A	107.6
H5A—C5—H5B	109.1	C1—C20—H20A	107.6
N1—C6—C11	111.57 (13)	C21—C20—H20B	107.6
N1—C6—C7	110.19 (13)	C1—C20—H20B	107.6
C11—C6—C7	107.37 (13)	H20A—C20—H20B	107.1
N1—C6—H6	109.2	C26—C21—C22	116.57 (19)
C11—C6—H6	109.2	C26—C21—C20	120.61 (18)
C7—C6—H6	109.2	C22—C21—C20	122.74 (18)
C8—C7—C6	109.52 (13)	C23—C22—C21	121.7 (2)
C8—C7—S1	108.36 (11)	C23—C22—H22	119.1
C6—C7—S1	114.05 (11)	C21—C22—H22	119.1
C8—C7—H7	108.3	C24—C23—C22	121.1 (2)
C6—C7—H7	108.3	C24—C23—H23	119.4
S1—C7—H7	108.3	C22—C23—H23	119.4
O3—C8—O4	124.95 (17)	C25—C24—C23	117.5 (2)
O3—C8—C7	124.37 (16)	C25—C24—C24A	121.7 (2)
O4—C8—C7	110.67 (14)	C23—C24—C24A	120.8 (2)
O4—C9—C10	111.7 (2)	C24—C24A—H24A	109.5
O4—C9—H9A	109.3	C24—C24A—H24B	109.5
C10—C9—H9A	109.3	H24A—C24A—H24B	109.5
O4—C9—H9B	109.3	C24—C24A—H24C	109.5
C10—C9—H9B	109.3	H24A—C24A—H24C	109.5
H9A—C9—H9B	107.9	H24B—C24A—H24C	109.5
C9—C10—H10A	109.5	C24—C25—C26	121.7 (2)
C9—C10—H10B	109.5	C24—C25—H25	119.1
H10A—C10—H10B	109.5	C26—C25—H25	119.1
C9—C10—H10C	109.5	C21—C26—C25	121.4 (2)
H10A—C10—H10C	109.5	C21—C26—H26	119.3
H10B—C10—H10C	109.5	C25—C26—H26	119.3
C16—C11—C12	118.10 (16)		
			
O2—S1—C1—C17	−49.60 (14)	S1—C7—C8—O3	−69.1 (2)
O1—S1—C1—C17	−179.61 (11)	C6—C7—C8—O4	−122.94 (15)
C7—S1—C1—C17	65.90 (13)	S1—C7—C8—O4	112.09 (14)
O2—S1—C1—C20	76.17 (13)	C8—O4—C9—C10	−74.8 (3)
O1—S1—C1—C20	−53.83 (13)	N1—C6—C11—C16	122.88 (17)
C7—S1—C1—C20	−168.32 (11)	C7—C6—C11—C16	−116.29 (17)
O2—S1—C1—C2	−167.63 (10)	N1—C6—C11—C12	−59.7 (2)
O1—S1—C1—C2	62.36 (12)	C7—C6—C11—C12	61.13 (19)
C7—S1—C1—C2	−52.13 (12)	C16—C11—C12—C13	0.6 (3)
C6—N1—C2—C3	157.61 (14)	C6—C11—C12—C13	−176.90 (17)
C5—N1—C2—C3	35.70 (18)	C11—C12—C13—C14	0.0 (3)
C6—N1—C2—C1	−75.69 (17)	C12—C13—C14—C15	−0.5 (3)
C5—N1—C2—C1	162.40 (14)	C12—C13—C14—C14A	179.4 (2)
C17—C1—C2—N1	−50.27 (17)	C13—C14—C15—C16	0.5 (3)
C20—C1—C2—N1	−177.16 (14)	C14A—C14—C15—C16	−179.5 (2)
S1—C1—C2—N1	67.73 (14)	C12—C11—C16—C15	−0.7 (3)
C17—C1—C2—C3	69.51 (19)	C6—C11—C16—C15	176.82 (17)
C20—C1—C2—C3	−57.4 (2)	C14—C15—C16—C11	0.2 (3)
S1—C1—C2—C3	−172.48 (14)	C18—O6—C17—O5	−7.4 (3)
N1—C2—C3—C4	−17.1 (2)	C18—O6—C17—C1	170.01 (17)
C1—C2—C3—C4	−140.19 (19)	C20—C1—C17—O5	−139.09 (19)
C2—C3—C4—C5	−7.5 (3)	C2—C1—C17—O5	97.1 (2)
C6—N1—C5—C4	−163.15 (16)	S1—C1—C17—O5	−17.4 (2)
C2—N1—C5—C4	−40.30 (19)	C20—C1—C17—O6	43.57 (19)
C3—C4—C5—N1	29.2 (3)	C2—C1—C17—O6	−80.20 (17)
C2—N1—C6—C11	−177.18 (13)	S1—C1—C17—O6	165.25 (12)
C5—N1—C6—C11	−59.59 (17)	C17—O6—C18—C19	84.3 (2)
C2—N1—C6—C7	63.65 (16)	C17—C1—C20—C21	66.2 (2)
C5—N1—C6—C7	−178.76 (14)	C2—C1—C20—C21	−169.65 (15)
N1—C6—C7—C8	−174.19 (12)	S1—C1—C20—C21	−56.66 (18)
C11—C6—C7—C8	64.12 (16)	C1—C20—C21—C26	115.4 (2)
N1—C6—C7—S1	−52.58 (15)	C1—C20—C21—C22	−67.9 (2)
C11—C6—C7—S1	−174.27 (11)	C26—C21—C22—C23	0.9 (3)
O2—S1—C7—C8	−70.91 (12)	C20—C21—C22—C23	−175.9 (2)
O1—S1—C7—C8	58.70 (13)	C21—C22—C23—C24	0.9 (4)
C1—S1—C7—C8	170.15 (11)	C22—C23—C24—C25	−1.8 (4)
O2—S1—C7—C6	166.85 (11)	C22—C23—C24—C24A	176.5 (2)
O1—S1—C7—C6	−63.55 (13)	C23—C24—C25—C26	0.8 (4)
C1—S1—C7—C6	47.91 (12)	C24A—C24—C25—C26	−177.5 (2)
C9—O4—C8—O3	−5.5 (3)	C22—C21—C26—C25	−1.9 (3)
C9—O4—C8—C7	173.31 (17)	C20—C21—C26—C25	175.0 (2)
C6—C7—C8—O3	55.9 (2)	C24—C25—C26—C21	1.1 (4)

### Diethyl 1-(4-methylbenzyl)-4-(4-methylphenyl)-2,2-dioxo-3,4,6,7,8,8a-hexahydro-1H-pyrrolo[2,1-c][1,4]thiazine-1,3-dicarboxylate (II) Hydrogen-bond geometry (Å, º)

**Table:**

*D*—H···*A*	*D*—H	H···*A*	*D*···*A*	*D*—H···*A*
C16—H16···O1^i^	0.93	2.57	3.406 (2)	150
C18—H18*B*···O1^ii^	0.97	2.40	3.333 (2)	161

Symmetry codes: (i) −*x*+1, −*y*+1, −*z*+2; (ii) *x*−1/2, −*y*+3/2, *z*−1/2.

## References

[bb1] Armenise, D., Trapani, G., Arrivo, V. & Morlacchi, F. (1991). *Farmaco*, **46**, 1023–1032. 1807288

[bb2] Armenise, D., Trapani, G., Stasi, F. & Morlacchi, F. (1998). *Arch. Pharm. Pharm. Med. Chem.* **331**, 54–58. 10.1002/(sici)1521-4184(199802)331:2<54::aid-ardp54>3.0.co;2-6 9525089

[bb3] Bruker (2009). *APEX2*, *SAINT* and *SADABS*. Bruker AXS Inc., Madison, Wisconsin, USA.

[bb4] Chitradevi, A., Athimoolam, S., Bahadur, S. A., Indumathi, S. & Perumal, S. (2011). *Acta Cryst.* E**67**, o2268. 10.1107/S1600536811031047 PMC3200855 22058923

[bb5] Chitradevi, A., Athimoolam, S., Bahadur, S. A., Indumathi, S. & Perumal, S. (2013). *Acta Cryst.* E**69**, o706–o707. 10.1107/S1600536813009148 PMC3648240 23723860

[bb6] Desiraju, G. R. & Sarma, J. A. R. P. (1986). *Proc. Indian Acad. Sci. Chem. Sci.* **96**, 599–605.

[bb7] Erker, T. (1998). *J. Heterocycl. Chem.* **35**, 1521–1526.

[bb8] Gnanaguru, K., Murthy, G. S., Venkatesan, K. & Ramamurthy, V. (1984). *Chem. Phys. Lett.* **109**, 255–258.

[bb9] Grandolini, G., Ambrogi, V., Perioli, L., D’Eramo, D., Bernardini, C. & Giampietri, A. (1997). *Farmaco*, **52**, 379–384. 9372589

[bb10] Groom, C. R., Bruno, I. J., Lightfoot, M. P. & Ward, S. C. (2016). *Acta Cryst.* B**72**, 171–179. 10.1107/S2052520616003954 PMC4822653 27048719

[bb11] Indumathi, S., Kumar, R. R. & Perumal, S. (2007). *Tetrahedron*, **63**, 1411–1416.

[bb12] Jones, W., Ramdas, S., Theocharis, C. R., Thomas, J. M. & Thomas, N. W. (1981). *J. Phys. Chem.* **85**, 2594–2597.

[bb13] Kitaigorodskii, A. I. (1973). *Molecular Crystals and Molecules*. New York: Academic Press.

[bb14] Koketsu, M., Tanaka, K., Takenaka, Y., Kwong, C. D. & Ishihara, H. (2002). *Eur. J. Pharm. Sci.* **15**, 307–310. 10.1016/s0928-0987(02)00014-3 11923063

[bb15] Krishnaiah, M., Jagadeesh Kumar, N., Bhaskar Reddy, D., Muralidhar Reddy, M., Soriano, M., Chen, Y.-S. & Narasinga Rao, S. (1995). *Acta Cryst.* C**51**, 2426–2428.

[bb16] Malinka, W., Kaczmarz, M., Filipek, B., Sapa, J. & Glod, B. (2002). *Farmaco*, **57**, 737–746. 10.1016/s0014-827x(02)01267-3 12385524

[bb17] Moriyama, H., Tsukida, T., Inoue, Y., Yokota, K., Yoshino, K., Kondo, H., Miura, N. & Nishimura, S. (2004). *J. Med. Chem.* **47**, 1930–1938. 10.1021/jm0304313 15055993

[bb18] Rai, V. K., Rai, P. & Thakur, Y. (2013). *Tetrahedron Lett.* **54**, 6469–6473.

[bb19] Rajni Swamy, V. (2016). PhD thesis, Madurai Kamraj University, India.

[bb20] Rajni Swamy, V., Müller, P., Srinivasan, N., Perumal, S. & Krishnakumar, R. V. (2013). *Acta Cryst.* C**69**, 412–415. 10.1107/S0108270113004812 23579718

[bb21] Sheldrick, G. M. (2008). *Acta Cryst.* A**64**, 112–122. 10.1107/S0108767307043930 18156677

[bb22] Sheldrick, G. M. (2015). *Acta Cryst.* C**71**, 3–8.

[bb23] Spackman, M. A. & Jayatilaka, D. (2009). *CrystEngComm*, **11**, 19–32.

[bb24] Spek, A. L. (2009). *Acta Cryst.* D**65**, 148–155. 10.1107/S090744490804362X PMC2631630 19171970

[bb25] Sugumar, P., Edayadulla, N., Ramesh, P., Ramesh, P. & Ponnuswamy, M. N. (2011). *Acta Cryst.* E**67**, o305. 10.1107/S1600536811000444 PMC3051777 21522994

[bb26] Westrip, S. P. (2010). *J. Appl. Cryst.* **43**, 920–925.

[bb27] Wolff, S. K., Grimwood, D. J., McKinnon, J. J., Turner, M. J., Jayatilaka, D. & Spackman, M. A. (2012). *Crystal Explorer.* University of Western Australia.

